# 

**Published:** 1906-02-03

**Authors:** 


### NOTICE TO CORRESPONDENTS.

All MSS., Letters, Books for Review, and other matters intended for the Editor should be addressed The Editor, "The Hospital," The Hospital Buildings, 28 & 29 Southampton Street, Strand, W.C.

The Editor cannot undertake to return rejected MS., even when accompanied by stamped directed envelope.

**Notice to Advertisers and Subscribers.**

All Advertisements, Orders for Copies of The Hospital, and Business Communications should be addressed to **THE MANAGER (Not to the Editor),** The Hospital, 28 & 29 Southampton Street, Strand London, W.C.

The Cost of Subscription, post free, is as follows:—Per week, 3½d.; per quarter, 3s. 3d.; per half-year, 6s. 6d.; per annum, 13s. Foreign and Colonial:—Per annum, 19s. 6d., payable, in all cases, in advance.

**NOTICE.—Vol. XXXVIII. of "The Hospital" April to September 1905), in handsome cloth binding, will shortly be on sale at the office of this Journal, price 10s. 6d. Cases for binding the Numbers contained in this Volume 2s. Index to Vol. XXXVIII., 3½d., post free.**

**The Monthly Part for February is now ready. Price 1s., by post, 1s. 3d.**

### Medical Appointments and Vacancies.

### VACANCIES.

Barnsley, Beckett Hospital.—House Surgeon.

Bradford Poor Law Hospital and Workhouse.—Resident Assistant Medical Officer.

Cancer Hospital, Fulham Road, London, S.W.—Senior House Surgeon.

Canterbury, Kent and Canterbury Hospital.—House Physician, also House Surgeon.

Cheltenham General Hospital.—Surgeon-in-Charge of Branch Dispensary.

City of London Hospital for Diseases of the Chest, Victoria Park, E.—Physician.

Hospital for Sick Children, Great Ormond Street, London, W.C.—Assistant Casualty Medical Officer. Also House Physician.

Leeds General Infirmary.—Resident Medical Officer.

Leicester Infirmary.—Assistant House Physician.

Margate, Royal Sea Bathing Hospital.—Resident Surgeon.

Poplar Hospital for Accidents, Poplar, E.—Assistant House Surgeon.

Queen Charlotte's Lying-in Hospital, Marylebone Road, N.W.—Assistant Resident Medical Officer for four months.

St. Mark's Hospital for Fistula and Other Diseases of the Rectum, City Road, E.C.—House Surgeon.

St. Mary's Hospital Medical School, Paddington, W.—Lecturer on Physics.

Sheffield Royal Hospital.—Honorary Dental Officer.

Sierra Leone, Princess Christian Hospital, Freetown, West Africa.—Medical Officer.

Sunderland Infirmary.—House Physician and Third House Surgeon.

### Books for Nurses

**Medical and Surgical Nursing.**

**A HANDBOOK FOR NURSES.**

By J. K. WATSON, M.D., M.B., C.M. New and Revised Edition. Profusely Illustrated. 5/- net, 5/4 post free.

**Dictionary of Terms used in Medicine, Midwifery, and Nursing.**

**THE NURSES' PRONOUNCING DICTIONARY.**

By HONNOR MORTEN. New Edition, Revised by Mary I. Burdett. 2/- post free.

This Dictionary has been used by Nurses for many years, and the fact that it has been recently revised and the phonetic pronunciations added by Miss M. I. Burdett should greatly enhance its value as a work of reference. Its size enables it to be carried comfortably in the apron pocket, which is a strong point in its favour.

**How to Bandage.**

**PRACTICAL GUIDE TO SURGICAL BANDAGING AND DRESSINGS.**

By WM. JOHNSON SMITH, F.R.C.S. 2/- post free.

A useful guide to Bandaging and Dressing, written especially for Nurses by a surgeon of great experience. The work is up to date, and the information thoroughly reliable.

**Anatomy and Physiology.**

**ELEMENTARY ANATOMY AND SURGERY FOR NURSES.**

By WILLIAM McADAM ECCLES, M.S.Lond., M.B., F.R.C.S., L.R.C.P. 2/6 post free.

**ELEMENTARY PHYSIOLOGY FOR NURSES.**

By C. F. MARSHALL, M.D., B.Sc., F.R.C.S. Crown 8vo, Illustrated, cloth. 2/- post free.

A valuable help to Physiology for Probationers.

**The Midwives Act Explained.**

**HOW TO BECOME A CERTIFIED MIDWIFE.**

By Dr. APPEL. 1/- net, 1/1 post free.

This book gives a very clear and complete explanation of the Midwives Act, and a copy should be in the possession of all Midwives and those nurses desirous of taking up the special branch.

**INSTRUCTIONS TO MIDWIVES.**

Compiled by the Manchester Midwives' Committee. Crown 8vo. cloth, 6d. net, 7d. post free.

Midwives will find this little work exceedingly useful as it explains simply and concisely what should be done in accordance with the rules of the Midwives Act.

**Medical Electricity.**

**MEDICAL ELECTRICITY AND THE LIGHT TREATMENT.**

By KATE NEALE, Sister in Charge Light Department, Guy's Hospital.

Cloth, profusely Illustrated, 2/6 net, 2/9 post free.

A practical book on a subject hitherto untouched from a nursing standpoint.

**THE EXAMINATION OF THE URINE.**

Compiled for the use of Nurses. By J. K. WATSON, M.D. M.B., C.M. 1/- net, 1/1 post free.

**How to Succeed at the C.M.B. Exam.**

**A COMPLETE HANDBOOK OF MIDWIFERY.**

By J. K. WATSON, M.D. 6/- net, 6/4 post free. Profusely Illustrated with 143 Diagrams.

This book has been **compiled in accordance with the requirements of the Central Midwives Board,** and will prove not only the best text-book for those entering for the next exam., but is so complete as to be of greatest value to the Qualified Midwife for immediate reference in cases of emergency.

**A Complete Text-Book of Medical, Surgical, and Monthly Nursing.**

**NURSING: ITS THEORY AND PRACTICE.**

By PERCY G. LEWIS, M.D., M.R.C.S., L.S.A. New Edition Profusely Illustrated. 3/6 post free.

**Surgical Work.**

**SURGICAL WARD-WORK AND NURSING.**

By ALEXANDER MILES, M.D.Edin. C.M., F.R.C.S.E. Second Edition. With particulars and Illustrations of the Instruments used in various Operations. 3/6 net, 3/10 post free.

**SURGICAL INSTRUMENTS AND APPLIANCES USED IN OPERATIONS.**

A Classified and **Illustrated** List, with Explanatory Notes. By HAROLD BURROWS, F.R.C.S. Ready Shortly. Limp cloth. 1/6 net, 1/8 post free.

This work will prove of the utmost service as a work of reference to all Nurses, as not only does it contain particulars of instruments but is fully illustrated so that they can be readily recognised.

**The Duties of a Nurse Explained.**

It is essential that anyone desirous of taking up Nursing should have some idea of the duties of a Nurse.

**NURSING HINTS TO PROBATIONERS ON PRACTICAL WORK.**

By ANNESLEY VOYSEY. 2/- net, 2/4 post free.

Contains full particulars of a Nurse's duties, and will prove of the greatest assistance to the Probationer.

**An Important New Work on Sanitation for Nurses.**

**SIMPLE SANITATION:**

**The Practical Application of the Laws of Health to Small Dwellings.**

By M. LOANE, Superintendent of District Nurses, Portsmouth. Limp cloth, 1/- net, 1/2 post free.

With an Introductory Chapter on Sanitary Legislation by Dr. A. Mearns Fraser, Medical Officer of Health, Portsmouth.

This work should prove of great practical assistance to Midwives and District Nurses, as it contains Chapters on Sanitary Legislation, Water, Air and Ventilation, Drainage and Disposal of Refuse, Household Management, Personal Hygiene, Disinfectants, &c.

**Nursing of Children.**

**NURSING OF SICK CHILDREN.**

By Dr. BURNET. 1/- net, 1/1 post free.

Nurses and Mothers will find this a most useful handbook.

**The Scientific Press, Ltd.**

*Complete Catalogue sent post free on application.*

**Publishers of Nursing Text-Books, Charts, and Case-Books of all Descriptions.**

**28 & 29 SOUTHAMPTON STREET, STRAND, LONDON, W.C.**

